# Temperature shifts associated with bat arousals during hibernation inhibit the growth of *Pseudogymnoascus destructans*

**DOI:** 10.1098/rsos.211986

**Published:** 2022-11-23

**Authors:** Ronny Forney, Gabriela Rios-Sotelo, Alexa Lindauer, Craig K. R. Willis, Jamie Voyles

**Affiliations:** ^1^ Department of Biology, University of Nevada, Reno, NV, USA; ^2^ Sierra Nevada Aquatic Research Laboratory, University of California, Santa Barbara, Mammoth Lakes, CA, USA; ^3^ Department of Biology, University of Winnipeg, Winnipeg, Manitoba, Canada

**Keywords:** arousals, bats, hibernation, *Pseudogymnoascus destructans*, white-nose syndrome

## Abstract

Temperature is a critically important factor in many infectious disease systems, because it can regulate responses in both the host and the pathogen. White-nose syndrome (WNS) in bats is a severe infectious disease caused by the temperature-sensitive fungus, *Pseudogymnoascus destructans* (*Pd*). One feature of WNS is an increase in the frequency of arousal bouts (i.e. when bat body temperatures are elevated) in *Pd*-infected bats during hibernation. While several studies have proposed that increased frequency of arousals may play a role in the pathophysiology of WNS, it is unknown if the temperature fluctuations might mediate *Pd* growth. We hypothesized that exposure to a high frequency of elevated temperatures would reduce *Pd* growth due to thermal constraints on the pathogen. We simulated the thermal conditions for arousal bouts of uninfected and infected bats during hibernation (fluctuating from 8 to 25°C at two different rates) and quantified *Pd* growth *in vitro*. We found that increased exposure to high temperatures significantly reduced *Pd* growth. Because temperature is one of the most critical abiotic factors mediating host–pathogen interactions, resolving how *Pd* responds to fluctuating temperatures will provide insights for understanding WNS in bats and other fungal diseases.

## 1. Introduction

**Figure 1.**
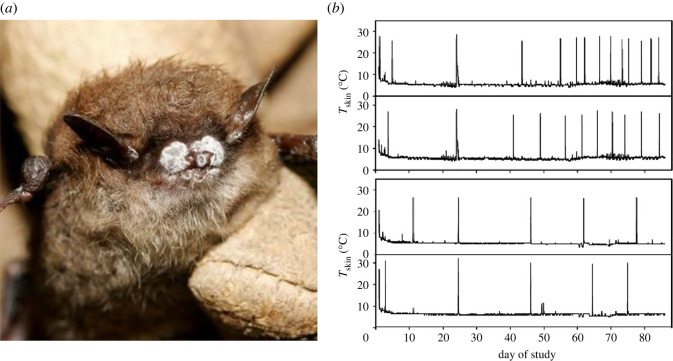
Bat infected with *Pseudogymnoascus destructans* (*Pd*), where *Pd* is visible (white) on the skin of the nose (*a*). Bat skin temperatures for bats experimentally over time since experimental infection with *Pd* (*b*). Increased skin temperatures represent an arousal from torpor when body temperatures are elevated during hibernation. Bats infected with *Pd* have more frequent arousals (top rows) compared with uninfected bats (bottom rows) as disease development progresses over time. Photo credit: Ryan von Linden. Figure 1*b* modified from Warnecke *et al*. [10].

Temperature is among the most important abiotic influences on ecological processes, including host–parasite interactions [1–3]. There are many ways that temperature can influence host–parasite interactions, but some of the most important include how temperature affects the ability of a host to defend itself and a pathogen's ability to colonize and reproduce within a host [1,2]. Temperature-sensitivity of pathogens is a key consideration for many disease systems, including those caused by fungal pathogens, which are increasingly threatening many host organisms [4,5]. White-nose syndrome (WNS) in bats is one example of a mammalian fungal disease that is strongly influenced by environmental conditions (e.g. temperature and relative humidity) [6–9]. This disease is caused by the cold-tolerant, invasive fungus *Pseudogymnoascus destructans* (*Pd*; [6–9]; figure 1*a*). To date, *Pd* has been detected on 20 bat species with evidence of WNS in 12 of these species, all of which hibernate in caves or mines across 35 states in the United States and seven Canadian provinces [11]. Several species have been listed, or are being considered for listing, as endangered or threatened in the United States and Canada because of WNS. Given the severity of disease-induced bat declines in these regions, WNS has garnered considerable attention among scientists, conservation and management agencies, and the general public [6–9,11].

The mechanism of pathogenesis is one aspect of WNS that has been particularly puzzling [12–15]. During hibernation, when bat body temperatures are depressed, *Pd* invades epidermal tissues of the muzzle, ears and wings of bats [12–15]. Signs of severe disease are frequently evident late in the hibernation season, when bats have been observed on the ground or flying outside hibernacula (possibly looking for food or water), severely emaciated, visibly covered in white fungus, and/or exhibiting sickness behaviours [9,16–18]. Upon examination, additional clinical signs of disease can include cutaneous lesions and other epidermal pathology (e.g. on wing tissues), reduced electrolyte concentrations, and depletion of fat stores, all of which suggest additional complexities in the pathophysiology of WNS [10,15,16,19].

In addition to alternations of bat physiological processes, several studies report disruptions of important hibernation behaviours in *Pd*-infected bats [10,20,21]. All mammalian hibernators, including bats, normally undergo episodes of periodic arousal during hibernation [22–24]. The biological significance of arousals is not fully understood (even in healthy bats and other hibernators), but several hypotheses have been proposed, including excretion of metabolic wastes, restoration of water balance, changing roost position and re-establishing immune function [15,24–28]. Some studies have provided experimental evidence that *Pd*-infected bats exhibit more frequent arousal bouts compared with uninfected controls, with increased frequency of arousal especially pronounced in the late stages of infection [10,21]. Thermogenesis for periodic arousals during hibernation requires a tremendous investment of energy, which suggests that metabolic costs of more frequent arousals could explain bat mortality from WNS [10,21]. However, *Pd*-infected bats also appear to have increased energy expenditure during torpor bouts throughout hibernation [15]. Increasing body temperatures is generally thought to facilitate host immune responses to pathogens [29], but at least one study has shown that some bats in remnant populations experiencing WNS-induced mortality do not exhibit increases in arousal frequency [30]. As such, the functional importance of increased bat arousals in WNS is not fully understood [15,29].

The temperature-sensitivity of *Pd* is an important component of WNS disease dynamics. A large body of work demonstrates that temperature plays a clear role in disease severity, host survivorship and recovery in wild bats in different habitats [31–35], influences seasonal disease dynamics [33,36] and mediates pathogen growth and pathogenesis in laboratory studies [37–39]. In addition, a growing number of field and modelling studies have confirmed the importance of environmental conditions on WNS impacts [40–42]. In *in vitro* studies, active growth of *Pd* occurs within a thermal range of 0 to 20°C [37–39] and greater than 81% relative humidity [43]. Verant *et al*. [37] reported a thermal optimum (*T*_opt_) for *Pd* growth from 12.5 to 15.8°C and a critical thermal maximum (*CT*_max_) between 19 and 21°C (although one study reported conidia could remain viable, but without active growth, at 37°C [38]). However, to date most empirical studies have examined *Pd* responses in stable temperature conditions [37–39,41], rather than in fluctuating temperatures that pathogen might encounter as bat body temperatures change throughout cycles of torpor and arousal. For example, during periodic arousals, the little brown bat (*Myotis lucifugus*) *T*_b_ increases to 20–22°C, or even higher (approx. 24–26°C) if exhibiting a fever response [10,21,27]. Moreover, arousals occur more frequently in bats with advanced WNS in the later stages of *Pd* infection [10,21], which means that *Pd* will experience greater variation in its environmental temperature and more prolonged exposure to temperatures outside its thermal optimum. Therefore, experimental research that simulates fluctuating thermal conditions (e.g. temperature shifts that are biologically relevant) may advance our understanding of *Pd*'s responses to temperature and thus disease development.

Understanding responses of *Pd* to fluctuating temperatures—decoupled from the potential physiological responses of hosts—will improve our understanding of *Pd* biology and the thermal conditions that may support growth and survival of *Pd* in the environment and exacerbate or attenuate disease. In this study, we generated a simple experimental design to investigate how simulating fluctuating temperature would affect *Pd* growth. We hypothesized that *T_b_* fluctuations typical of those occurring during frequent periodic arousals throughout hibernation in infected bats (e.g. from approx. 8°C to approx. 25°C), would be sufficient to alter *Pd* growth patterns in the absence of any other effects of host biology (e.g. immune response). To test this hypothesis, we quantified *Pd* growth *in vitro* in high and low frequencies of temperature fluctuations, similar to those exhibited by hibernating bats that are susceptible to WNS (*Myotis lucifugus*; [18]).

## 2. Methods

### 2.1. Culturing and experimental set-up

We obtained a *Pd* culture from American Type Culture Collection (*Geomyces destructans* number ATCC MYA-4855), which was originally isolated from the wing of a little brown bat (*Myotis lucifugus*) in Williams Hotel Mine, Ulster County, New York, USA, in 2008 [7,8]. We revived the isolate by slowly warming the culture to room temperature (approx. 21–22°C) in a water bath (per ATCC manufacturer's instructions). We transferred the *Pd* to two Sabourand's dextrose agar (SDA) plates and incubated it at 15°C under 24 h darkness [37] until we initiated the temperature experiment.

### 2.2. Temperature experimental set-up

To harvest conidia for our experiment, we grew the cultures for 6–8 weeks on SDA agar plates or to when mycelia covered approximately 75% of the plates [37]. We collected conidia by flooding the plates with 5 ml 0.5% 1× phosphate buffered saline with Tween 20 (PBST) and allowed it to sit for 5 min [44]. We then drew off 5 ml from the agar plate, counted conidia concentrations using a haemocytometer (Hausser Scientific, Horsham, PA, USA). We diluted the solution to a concentration of 2 × 10^4^ conidia per 1 ml. We then inoculated 50 µl of the solution containing conidia into 30 wells in *N* = 8 Falcon 96-well, non-treated, flat-bottom microplates (Fisher Scientific, Waltham MA, USA). We also added 100 μl of Sabourand's dextrose solution media (for which we used the same methods as for SDA [37] but we omitted agar) to each well for all plates. To provide a negative control, we transferred the PBST solution containing *Pd* conidia to a 50 ml conical and submerged it in 100°C water for 10 min. We then added 50 µl of PBST solution that contained heat-killed *Pd* conidia and 100 µl of media to 30 negative control wells. We placed parafilm around the plates to maintain a tight seal on the lid and then incubated the plates in one of four temperature conditions and 80% humidity within incubators (Isotemp refrigerated incubators, Thermo Fisher, Waltham MA, USA) for 36 days.

The temperature treatments included: (i) constant 8°C, a constant temperature within the thermal breadth for *Pd* growth, (ii) constant 25°C, comparable to the skin temperature of hibernating bats during arousals [10], (iii) fluctuating between 8 and 25°C every 16 days, simulating the *T_b_* for ‘low-frequency arousal’ (uninfected, control) bats, and (iv) fluctuating between 8 and 25°C every 7 days, simulating the *T_b_* for ‘high-frequency arousal’ (i.e. infected bats with advanced WNS; figure 1*b*; [10]). For the fluctuating temperature treatments, the experimental plates were exposed to higher temperature conditions for 1 hour, which is the approximate duration of arousal for multiple bat species [10,45]. Over the course of the experiment, we inspected the wells for contamination using a light microscope, seated the plates in a microplate reader (BioTek ELx800 spectrophotometer using the 540 nm filter), and recorded optical density (OD) every 3 days up to 36 days.

### 2.3. Statistical analysis

We assessed the difference in *Pd* growth over time among different temperature treatments using nonlinear mixed effects models with the ‘nlme’ package v. 3.1.131.1 [30] in R v. 3.4.3 [46]. We selected a three-parameter logistic function because logistic growth curves are classically used to model the exponential and stationary phases of microbial growth *in vitro*. A nonlinear modelling approach allows for meaningful parameter estimates of culture stationary phase (i.e. asymptote), the time at which cultures are in exponential growth phase halfway to stationary phase (i.e. inflection point) and a scale parameter that determines the steepness of the growth curved. We corrected for initial conidia inoculation and media colour by subtracting OD values of heat-killed controls from OD values of wells containing *Pd*. Due to an error in pipetting, we omitted two wells in one of the plates from our analyses. We then built models using the adjusted mean OD per plate per day (*N* = 8 plates per temperature treatment).

We compared model fits with analysis of variance (ANOVA) and likelihood ratio tests for nested models, and we selected models using Akaike information criteria (AIC) and principles of parsimony following Pinheiro *et al.* [45]. *Pd* incubated at a constant temperature of 25°C did not follow a logistic growth pattern (i.e. there was no *Pd* growth at this temperature) and we therefore excluded this temperature treatment from the final analysis. Our final model included fixed effects of temperature treatment on all three logistic growth parameters and the random effect of plate on the inflection point and scale parameters. We added an identity variance structure and an autoregressive moving average correlation structure to the model to address heteroscedasticity [47]. This approach allowed us to address two interrelated issues. First, heteroscedasticity is a common statistical issue of unequal variance, which is a common assumption for basic statistical models. Second, when using mixed models for analysis, it is important to consider the covariance structure. An autoregressive structure can occur with increasing time. Because we used a repeated measures experimental design, this model provided a good option for data analysis. We used an *F*-test to determine the significance of the temperature treatment fixed effect.

## 3. Results

**Figure 2.**
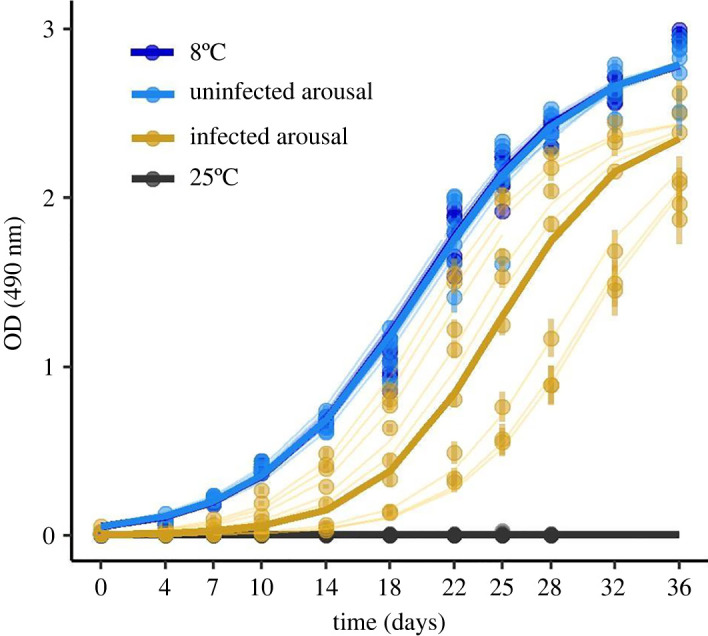
Exposure to increased frequency of simulated arousal bouts decreases growth of *Pseudogymnoascus destructans* (*Pd*) *in vitro*. *Pd* cultures grown under thermal conditions that simulate low-frequency arousal of uninfected bats (light blue) do not differ from *Pd* grown under an optimal stable temperature of 8°C (dark blue). *Pd* growth is reduced under high-frequency arousal exhibited by infected bats at the late stages of disease development (yellow). *Pd* did not grow under constant exposure to 25°C, a body temperature of euthermic bats (black). Heavy lines represent model fits by temperature treatment group; light lines represent model fit by plate; points and error bars represent means and standard error of wells per plate.

Exposure to different temperature treatments altered patterns of *Pd* growth *in vitro* (nonlinear mixed effects model, d.f. = 6, *F* = 13.1, *p* < 0.0001; figure 2). Specifically, when we incubated *Pd* in thermal conditions that simulated ‘high-frequency arousal’ (i.e. infected bats with advanced WNS), growth differed significantly from cultures incubated at 8°C (table 1). By contrast, *Pd* grown under conditions that simulated ‘low-frequency arousal’ (uninfected, control) bats did not differ from *Pd* growth when incubated at 8°C (table 1). We did not observe any growth in *Pd* cultures incubated at a stable temperature of 25°C. Overall, the lower parameter estimates for the asymptote, inflection point and scale parameters for *Pd* growth indicated that there was decreased total growth and a slower growth rate the ‘high-frequency arousal’ (i.e. infected bats with advanced WNS) temperature conditions (table 1).

**Table 1.** Parameter estimates for logistic growth models of *Pd* grown under different warming frequencies that simulate the arousal bouts of uninfected or infected bats, as compared with *Pd* grown at bat torpor body temperatures (8°C).

group	asymptote ± s.e.	*p*	inflection point ± s.e.	*p*	scale ± s.e.	*p*
8°C	2.76 ± 0.05	<0.0001	19.17 ± 0.89	<0.0001	4.78 ± 0.15	<0.0001
low-frequency arousal	2.78 ± 0.07	00.590	19.19 ± 1.26	0.984	4.88 ± 0.22	0.654
high-frequency arousal	2.53 ± 0.07	<0.0001	25.03 ± 1.26	0.001	3.90 ± 0.23	<0.0001

## 4. Discussion

Understanding the influence of environmental conditions on pathogen growth and reproduction is important for resolving mechanisms of pathogenesis, transmission and ecology in infectious disease systems [1–3]. For WNS in bats, previous studies have quantified *Pd* growth across a range of stable temperature conditions to characterize its fundamental thermal niche [37–42]. However, investigating how *Pd* responds to fluctuating temperatures that simulate conditions during arousals from torpor throughout hibernation could provide additional insights and help resolve several outstanding questions about WNS in multiple bat species.

We hypothesized that exposing *Pd* to fluctuating temperatures would inhibit *Pd* growth. We used a simple experimental design in which we expose *Pd* to temperature regimes simulating the low-frequency arousals of healthy bats and the higher frequency arousals of infected individuals [10]. Our results suggest that, in the absence of variation in other factors, more frequent exposure to high temperatures alone (i.e. those similar to patterns of body temperature shown by infected bats) significantly reduced *Pd* growth. While our treatments of increased temperature fluctuations slowed *Pd* growth, it is worth noting that they did not fully inhibit growth as compared with our high-temperature treatment (i.e. *T_a_* = 25°C). Our study focused on *Pd* growth *in vitro* and used a simplistic pattern of body temperature fluctuation to design our treatment regime. Therefore, our results do not fully account for all the factors that may influence *Pd* replication *in vivo* and in wild bat populations. Nevertheless, taken together, our results suggest that patterns of hibernating *T_b_* that fluctuate relatively quickly tend to reduce growth of *Pd,* which could have implications for hibernating bats and understanding WNS.

While our results suggest the possibility of temperature-mediated attenuation of *Pd* growth *in vitro*, some available evidence suggests that this may not translate into protection for susceptible bats *in vivo*. For example, Warnecke *et al*. [10] observed a progressive increase in *Pd* proliferation, even as arousal frequency increased in the terminal stages of disease development. Additionally, more recent studies have found that some bat species (as well as individual bats that survive *Pd*-infection) do not appear to have increased frequencies of arousal bouts when infected with *Pd* [26,30]. Nevertheless, for individual bats, populations and species that exhibit increased frequencies of arousal, and/or in those that show a fever response to *Pd*-infection [27], the possible inhibition of *Pd* due to bat body temperatures is an intriguing possibility to explore.

Understanding *Pd* growth *in vivo*, and/or in conditions that more closely match the ecological contexts experienced by wild bats, will require additional research. For example, future research in *in vitro* experiments could better simulate the alterations of bat body temperatures during torpor and arousal bouts. Such experiments could include variation in warming and cooling rates and variation in *T_b_* between healthy and infected individuals, including higher *T*_max_ temperatures exhibited by febrile bats during infection, adjusting the duration of time maintained at optimal and suboptimal temperatures, and mimicking normal torpor body temperatures of different bat species. Additional research should also examine *Pd* growth incorporating additional aspects of bat biology, such as bat defences (e.g. immune and microbiome factors), behaviours (e.g. grooming behaviours), and other host factors that probably influence *Pd* growth and the manifestation of disease.

Temperature is a critically important factor in many infectious disease systems because it can regulate both the host and the pathogen [1–3]. Our findings reveal an important, but perhaps underestimated, feature of *Pd* temperature sensitivity that could inform our understanding of WNS disease dynamics. Specifically, for bats with WNS the effects bat body temperatures have been a central focus of investigations on pathogenesis and pathophysiology [31–36], but the question of how temperature fluctuations may concomitantly alter *Pd* growth deserves further investigation. Ultimately, additional research of environmental constraints on pathogen growth can provide valuable insights into pathophysiology, transmission and spread, and the disease ecology of WNS and other devastating fungal diseases. Resolving such questions will lead to an improved understanding of infectious disease dynamics and may also facilitate conservation and management efforts.

## Data Availability

Data are available from the Dryad Digital Repository: https://doi.org/10.5061/dryad.sxksn036k [48]. Supplementary material is available online [49].
